# How Does Financial Development Affect Reductions in Carbon Emissions in High-Energy Industries?—A Perspective on Technological Progress

**DOI:** 10.3390/ijerph16173018

**Published:** 2019-08-21

**Authors:** Yingying Zhou, Zhuoqing Fang, Nan Li, Xueyan Wu, Yuehan Du, Zonghan Liu

**Affiliations:** 1 School of Management, China University of Mining and Technology, Xuzhou 221116, China; 2 Department of Economics, Jiangsu Normal University Kewen College, Xuzhou 221116, China

**Keywords:** high-energy industries, carbon emission reduction, financial development, technological progress, panel-threshold effect

## Abstract

Climate change has made countries around the world realize the importance of reducing carbon emissions. Reductions in carbon emissions needs the support of policy, technology, and financial capital. The single/double/three-threshold model is used here with data from China to study the different impact of financial development in carbon emissions in high-energy industries when the threshold variables are in different intervals. The results show that when loan size is the core explanatory variable, and research and development (R and D) expenditure and energy structure are the threshold variables, the loan size variable has a significant effect on emission reductions in high-energy industries, and this effect is strengthened with increases in R and D expenditure and decreases in the proportion of energy from coal. Taking energy intensity as the threshold variable, the relationship between loan size and carbon dioxide emissions is V-shaped. With economic structure as the threshold variable, loan size has a significant effect on emissions reduction when the proportion of industrial added value in high-energy industries is low. When using foreign investment as the core explanatory variable, R and D expenditure, energy consumption intensity, and industrial structure are threshold variables. The impact of foreign investment on carbon dioxide emissions is negative, but when the threshold variable is within different intervals, this negative impact differs. With stock market value as the core explanatory variable, and R and D expenditure and energy structure as the threshold variables, the stock market value can promote reductions in carbon emissions, but when R and D expenditure and the proportion of coal consumption is high, stock market value has no significant effect on emissions reduction. When energy consumption intensity is the threshold variable, the relationship between stock market value and carbon dioxide emissions is V-shaped.

## 1. Introduction

As the largest developing country, China has made continuous efforts to save energy and reduce emissions, and has been playing an increasingly important role in promoting environmental improvement. During the period of the 12th Five-Year Plan, China achieved remarkable results in saving energy and emissions reductions from 2011 to 2015. For example, Energy consumption per unit GDP (i.e., energy intensity) in China decreased by 18.4%. Moreover, emissions with a chemical oxygen demand (COD), as well emissions of ammonia nitrogen, nitrogen oxides, and sulfur dioxide decreased by 12.9%, 13%, 18.6% and 18%, respectively, and emissions of other pollutants also decreased. The moves to emissions reductions have also helped to alleviate energy shortages, to readjust China’s industrial structure, and to improve the environment. The 13th Five-Year Plan for Comprehensive Work on Energy Saving and Emission Reduction has set a series of emission reduction targets. These include a proposal for total annual energy consumption to be limited to the equivalent of 5 billion tons of standard coal by 2020; in terms of energy consumption intensity, the target is to reduce energy consumption per 10,000 yuan of GDP by 15% by 2020 compared with that in 2015; and in terms of emissions, it proposed to limit COD emissions to 21 million tons by 2020, and to limit emissions of ammonia nitrogen to 2.07 million tons, sulfur dioxide to 15.8 million tons, and nitrogen oxides to 15.74 million tons, which are 10%, 10%, 15%, and 15% lower than in 2015, respectively. In terms of energy consumption structure, is the Plan proposed that by 2020, the proportion of coal in energy consumption will be reduced to less than 58%, and the proportion of natural gas and non-fossil energy will be increased to 10% and 15%, respectively. Furthermore, it aims to optimize the industrial structure by speeding up the development of service industries and strategic emerging industries, such that their proportion of added value to GDP will rise to 56% and 15%, respectively, by 2020, and by accelerating the development of green low-carbon industries, to make them the pillar industries of China’s economic development, with a total output of 100 billion yuan by 2020.

Chen Shiyi (2011) [1] made a study of China’s specific situation. Since reform and opening up, China’s industrial added value accounts for about 40% of GDP. However, industrial carbon dioxide emissions account for about 85% of the total, and peaked at over 90%. From this point of view, with the industrial sector has the greatest potential for reductions in carbon emissions. Economic growth is tightly linked to energy consumption, and in China the proportion of energy consumption that comes from fossil fuels is currently about 70%, with coal accounting for the highest proportion of this. This generates large amounts of carbon dioxide and, while initially promoting economic development, the excessive carbon dioxide emissions will ultimately lead to environmental deterioration, which in turn will constrain further economic development.

China’s economic structure has obvious energy consumption characteristics: firstly, China’s total carbon dioxide emissions are comparatively large; secondly, China’s energy consumption per unit GDP is relatively high; and, thirdly, China’s carbon dioxide emissions per unit GDP are comparatively large. In order to change China’s carbon emissions, it will be necessary to optimize its industrial structure and improve its energy consumption structure. At present, China’s economic development has entered the “new normal”; the speed of development of resource-based industries with high energy consumption and high emissions has gradually decreased, the industrial structure has been improved, and the growth rate in energy consumption has slowed remarkably. Nonetheless, China’s energy consumption is still growing rapidly. A shortage of resources and environmental problems are bottlenecks restricting China’s sustainable development, and the task of saving energy, and thereby reducing emissions, is arduous. The coordinated development of the economy, society, and the ecological environment is required. Reducing carbon emissions is fundamental to alleviating current economic and environmental problems. A low-carbon economy can be created to address climate change.

In its 2009 report *Global Green New Deal*, the United Nations Environment Programme (UNEP) pointed out that the misallocation of large amounts of capital was the root cause of multiple global crises; that is, excessive capital had been invested in the exploitation and utilization of non-renewable energy, while insufficient capital had been invested in research and development on renewable energy. Finance has the function of capital allocation. Guiding financial capital to invest more in clean energy development and low-carbon technology plays an important role in reducing carbon emissions. To achieve a low-carbon economy, there is an urgent need to fully understand the relationship between financial resources, carbon emissions, and economic development, and to study the impact of financial development on reductions in carbon emissions in high-energy industries. Capital allocation needs to be guided not only to low-carbon industries but also to the reduction of carbon emissions in high-energy industries.

The economic development had the characteristics of high-energy consumption and high emissions under the traditional economic development model of China, which has caused great pressure on China’s resource endowment and ecological environment for a long time. At present, the contradiction between China’s economic development and the environment has become increasingly prominent. The acceleration of industrialization and urbanization has led to an increase in energy consumption. The increase in carbon dioxide emissions leads to a decline in environmental quality, thereby reducing the environmental feedback to the economy, which will constrain economic development and put greater pressure on China’s economic transformation and carbon emission reduction. Industry is an important area of energy consumption and carbon dioxide emissions in China. The transformation and upgrading of high-energy industries is particularly important for the development of low-carbon economy. And the development of low-carbon economy requires the support of financial funds. For the study of the impact of financial development on carbon emission reduction, scholars at home and abroad have studied more about the linear relationship between the two. Moreover, there are few studies on the impact of financial development on carbon emission reduction in specific industries. It is necessary to conduct research on the impact of financial development on carbon emissions reduction in a specific industry, such as high-energy industries, which account for a large proportion of carbon dioxide emissions. Therefore, based on the perspective of technological progress, paper measures the impact of financial development on carbon emission reduction in high-energy industries from various financing modes, such as credit market, foreign direct investment, and stock market, and analyzes the different impacts of financial development on carbon emission reduction in high-energy industries at different stages.

## 2. Literature Review

Energy shortages and environmental deterioration have aroused international concern. Saving energy, reducing carbon emissions and developing a low-carbon economy have become the broad consensus of all countries. However, this requires large-scale financial support. Domestic and foreign research on the impact of financial development on carbon emissions has focused on the promotion of technological innovation (which, in turn, is associated with reductions in carbon emissions), and how financial development can channel investment towards emissions reduction.

### 2.1. Research on the Relationship between Financial Development and Reductions in Carbon Emissions

Many countries are seeking to develop a low-carbon economy, but this requires funding. Scholars from different countries have studied the relationship between financial development and reductions in carbon emissions from different angles. There are three possible views on the relationship between them: financial development promotes increases in carbon emissions; financial development has a significant role in reducing carbon emissions; the relationship between financial development and carbon emissions is nonlinear. 

Some scholars have found a positive correlation between financial development and carbon emissions (Guo junjun et al., 2012 [2]; Xiong Ling and Qi Shaozhou, 2016 [3]). Zhang (2011) [4] studied the impact of China’s financial development on carbon emissions from multiple perspectives, and found that financial development is an important factor leading to increases in carbon emissions; the scale of financial intermediaries had the most significant impact. Chen Biqiong and Zhang Liangliang (2014) [5], used GMM (generalized method of moments) to study the impact of financial scale, financial efficiency and other factors on China’s carbon emissions. The results show that growth of financial scale and greater financial efficiency lead to increases in carbon emissions. 

Other scholars have found a negative correlation between financial development and carbon emissions. They believe that financial development can play a positive role in reducing carbon emissions (Tamazian et al., 2009 [6]; Ashina et al., 2012 [7]; Gu Hongmei and He Bin, 2012 [8]; Du Li et al., 2012 [9]; Halimanjaya, 2015 [10]; Zhu Wanli et al., 2015 [11]; Hao Liya, 2016 [12]). Shahbaz et al. (2013) [13] looked at data for Malaysia over the period 1971–2011 and found a long-term stable equilibrium relationship between financial development and carbon emissions, whereby financial development was conducive to reducing carbon emissions. Wang Qian et al. (2012) [14] used a fixed effect model to test the relationship between financial support and carbon productivity in 14 EU countries over the period 2005–2009. Financial support was found to have a significant impact on reductions in carbon emissions, and financial development contributed to the development of a low-carbon economy. Chen Fusheng (2014) [15] used GMM to study the impact of credit balance and bond market financing on green industry output value and carbon emissions. The results show that credit support and bond market financing have a positive effect on the development of a low-carbon economy and reductions in carbon emissions; of the two, credit support plays a stronger role in regulating the economic structure. 

Finally, some scholars believe that the relationship between financial development and carbon emissions is not simple and linear. Rather, they point out to an inverted U-shaped relationship (Chen Xin and Liu Ming, 2015 [16]; Li Ting and Li Wenxing, 2016 [17]). Zhang Yuejun, and Li Weikang (2016) [18] used a state space model to study the dynamic impact of financial development on carbon emissions in Beijing. In the sample interval, the impact of financial development on carbon emissions in Beijing had time-varying characteristics, and its impact could be either positive or negative; moreover, the contribution of financial development to carbon emissions was significantly less than that of economic development. Yan Chengliang et al. (2016) [19] used panel data from 30 provinces in China over the period 1997–2012 to study the impact of financial development on carbon emission intensity. Credit scale had an inverted U-type relationship with carbon intensity, and there is a U-shaped relationship between the scale of foreign direct investment (FDI) and carbon emission intensity in China. The sum of all loans in financial markets, competition within the financial industry, and marketization of credit fund allocation all have a negative impact on carbon emission intensity in China.

There are many ways to reduce carbon emissions, such as using less air conditioning and heating, driving less, flying less, and so on, in daily life. Most carbon emissions come from industrial production. Reducing carbon emissions in industrial production can reduce the scale of industrial production or promote carbon emission reduction in industrial production through energy-saving and loss-reduction technologies, which need financial support. In the process of carbon emission reduction, it is particularly necessary to pay attention to carbon emission reduction in industrial industries, which account for the majority of carbon emissions. However, it is not advisable to reduce industrial production blindly. The development of society cannot be separated from industry. Therefore, in the process of industrial development, energy saving and emission reduction need technical support, as well as the development of low-carbon economy, and circular economy etc., all need the support of financial development. The *United Nations Framework Convention on Climate* differs from developed and developing countries in terms of their obligations and procedures for fulfilling them. The Convention requires developed countries, as major emitters of greenhouse gases, to take concrete measures to limit greenhouse gas emissions and to provide funds to developing countries to cover the costs they need to meet their obligations under the Convention. The Convention establishes a financial mechanism for providing financial resources and technology to developing countries to enable them to fulfil their obligations under the Convention. The above scholars have studied the relationship between financial development and carbon emission reduction. Different scholars have different research samples and perspectives, so their conclusions are different. The main reasons that lead to different conclusions are the inconsistency of the research sample interval chosen by scholars, the inconsistency of the specific representative indicators selected, and the different countries studied. In the process of developing low-carbon economy, the impact of financial development on low-carbon economy cannot be ignored. In this process, financial development promotes technological progress and then has an impact on carbon emission reduction. In Section 2.2, this paper mainly explains scholars’ research in this area.

### 2.2. Research on Whether Financial Development Promotes Technological Innovation, Which Can Lead to Reductions in Carbon Emissions

The key to carbon emission reduction lies in technological progress and innovation. Technological progress is the most important driving force for improving energy efficiency (Fisher-Vanden et al., 2004 [20]; Li Lianshui and Zhou Yong, 2006 [21]; Dong Feng et al., 2010 [22]; Pan Xiongfeng et al., 2017 [23]). Technological progress requires funding. The financial sector can guide the flow of resources towards the achievement of high energy efficiency because of its role in resource allocation (Allen and Gale, 1997 [24]; Kyle, 1984 [25]; Merton, 1987 [26]). In order to promote reductions in carbon emissions, it is necessary to promote increases in energy efficiency and to change the structure of energy consumption. The application of ‘new energy’ within a low-carbon economy and of innovative emissions reduction technology cannot be separated from the issue of finance (Linares and Perez-Arriaga, 2009 [27]; Gouvello, 2010 [28]). Therefore, some scholars focus on the relationship between financial development and carbon emissions from the perspective of technological progress. They believe that financial development can give priority to projects and enterprises involving technological progress by giving full play to its resource allocation function, so as to achieve effective utilization of resources (Birdsall and Wheeler, 1993 [29]; Hanson and Laitner, 2004 [30]; Shahbaz et al., 2013 [13]). Beck et al. (2000) [31] used a dynamic panel model to conduct empirical research. The results show that financial development promotes economic growth not only because it increases the capital stock, but also because it improves the economy’s total factor productivity, that is, financial development can promote economic growth by promoting technological innovation, which is conducive to reducing carbon emissions. Hanson and Laitner (2004) [30] conducted research using an industrial growth assessment model, and concluded that the implementation of investment policies that guide technological progress can ensure reductions in carbon dioxide emissions, which is conducive to the economic growth of the United States, indicating that the United States needs to invest a large amount of money in low-carbon technology to reduce its carbon emissions. 

Some scholars believe that financial development can help improve energy utilization, thereby curbing environmental degradation and promoting reductions in carbon emissions (Fuente and Marin, 1995 [32]). Tamazian et al. (2009) [6] selected panel data for China, Russia, Brazil, and India over the period 1992–2004 to study the impact of financial development on carbon emissions, and then tested their model empirically on a dataset that included the United States and Japan. It was found that financial development played an important role in reducing carbon emissions. Thus, direct investment in technological innovation was conducive to improving energy utilization, and thereby restraining environmental degradation and promoting low-carbon economic development.

### 2.3. Research on the Channeling of Finance to Emissions Reduction

Financial development plays an important role in capital guidance, resource allocation and technological progress, and can promote reductions in carbon emissions to some extent. As a trend in economic development, a low-carbon economy needs financial support and innovation in the financial sector. Scholars have elaborated on the channels for providing financial support for emissions reductions from various perspectives (Harunaa Gujba et al., 2012 [33]; Cao Junxin and Yao Bin, 2014 [34]; Halimanjaya, 2015 [10]). They have assessed whether financial institutions can be encouraged to actively carry out carbon finance business, have explored the development of the Chinese banking sector’s carbon finance business, and focused on enhancing policy support for the development of and innovation in the carbon finance business (Zhou Yu, 2010 [35]; Peng Jiangbo and Guo Qi, 2010 [36]; He Jianqing and Liu Rixing, 2012 [37]; Zhang Zhaoguo et al., 2013 [38]; Chen Xiaolong and Liu Xiaobing, 2013 [39]; Wang Yao and Wang Wentao, 2014 [40]; Ding Xin et al., 2014 [41]). 

Commercial banks, as the main participants in carbon financial transactions, should raise awareness of how the financial sector can promote environmental protection, actively develop green credit business, increase the availability of credit for investment in as well as research and development in energy and emission reduction technologies, reduce the availability of credit for industries with high energy consumption and excess capacity, and provide credit through financing and intermediary services to support the development of a low-carbon economy (Yang Qu, 2011 [42]; Zhang Jinshan, 2013 [43]; Cao Junxin and Yao Bin, 2014 [34]; Zhang Jihong et al., 2014 [44]). Gao Yan and Wang Huitong (2010) [45] suggested that the environmental risk assessments conducted by credit businesses needed to be strengthened. Through the identification and pre-assessment of environmental risk (high energy consumption and high pollution), some loans can be screened out, and environmental risk monitoring should be strictly implemented as part of the loan conditions. While focusing on the development of green credit business, commercial banks should develop their own specialties, expand intermediary business, provide carbon finance, trust, financial advisory services and other services to customers, and tap the potential of the carbon finance market (Li Ping, 2013 [46]). 

Rory Sullivan et al. (2013) [47] explored how to finance low-carbon cities and the opportunities, risks, and barriers that exist. The study concluded that these risks and obstacles can be alleviated to a certain extent by reducing the cost of low-carbon economic development through government support, government-enterprise cooperation and technological innovation. Yasuko Kameyama et al. (2015) [48] proposed that finance has become one of the important topics in climate change negotiations in recent years. Developing a low-carbon economy in Asia will require about US$1250–1490 billion in investment annually. At present, public investment is much lower than expected. The authors believed that if Asian countries could reach a consensus, more than half of the low-carbon economic investment could be achieved by public investment, while the rest would depend on private investment. 

Based on the research by domestic and foreign scholars, there are still directions for further research in China. For example, it is necessary to study the impact of financial development on carbon emissions in specific industrial sectors, such as high-energy industries, which account for a large proportion of carbon emissions. Based on the data on high-energy industries in China, the present study is a quantitative analysis of whether financial development has a threshold effect on the carbon emissions of the high-energy industrial sector.

## 3. The Theoretical Foundation of Financial Development Effect on Reductions in Carbon Emissions

Scholars have studied financial development from different perspectives. Goldsmith (1959) [49] was the first to propose the concept of financial development. In the book *Financial Structure and Development*, the essence of financial development was summarized as a change in financial structure, and financial structure was determined by the size of financial institutions and the stock of financial instruments. Goldsmith pointed out that financial development affects economic growth. Unlike Goldsmith’s structural perspective, Bodie and Merton (1995) [50], in their financial function theory, took a functional perspective and pointed out that a sound financial system is conducive to accelerating capital accumulation, directing savings to investment, optimizing resource allocation, and promoting healthy and sustainable economic development. Levine et al. (2002) [51] believes that financial development refers to the improvement of the financial system and the development of the financial industry. It is a global concept, where financial development is an increase in both quantity and quality, and qualitative improvement refers to the improvement of the financial system. With the enrichment and strengthening of financial functions, the continuous improvement of the financial system is beneficial to promoting the optimal allocation of financial resources and promoting the common development of finance and the economy. 

The financial policy transmission mechanism refers to the process of using financial policy instruments to achieve first a set of intermediate objectives, such that the ultimate policy objectives can be realized. In relation to emissions reductions, financial policy generally takes the form of instruments, such as credit and preferential interest rates. By adjusting energy prices, we can reduce energy consumption, improve energy efficiency, promote economic growth, optimize industrial structure, and meet carbon reduction targets. On the one hand, credit instruments can help to rationalize energy prices and make up for any price difference between low-carbon clean energy and high-carbon energy. Examples include low-interest loans and extended loan periods for the funding of the development of clean energy, research and development in energy-saving equipment or alternatively high interest rates and reduced credit for high-energy and high-emission enterprises. On the other hand, the preferential interest rates can reduce the cost of clean energy, increase the environmental cost of high-carbon energy, indirectly guide the reductions in the price of clean energy, and enhance the market competitiveness of low-carbon energy. These are intermediate goals; the ultimate policy objective, of substantial reductions in carbon emissions, is achieved through the substitution of factors caused by these energy price changes.

Financial support is the basis for enterprises to reduce their carbon emissions. If there is no funding, it is difficult to guarantee technological transformation, low-carbon technology research and development, and emission-reduction projects. Therefore, we must give full play to the financial financing mechanism. There is a very large potential market for the development of low-carbon energy, and it has good prospects. However, its financing has the characteristics of large capital demand, high risk, a long payback period and complicated management. Therefore, the financing mechanisms will need a wide range of sources. Governments, financial institutions, enterprises, and residents will all be involved in the provision of comprehensive financing for energy conservation and emissions reductions. The financing for reducing carbon emissions, on the one hand, can absorb idle social funds and deploy them to areas such as clean energy development, low-carbon technology research and development, energy conservation and emission reduction projects; on the other hand, financial intermediation, through information consultation, technology integration and project management, can provide specialist services for energy-saving projects and enterprises, and optimize the financing environment.

The capital market has a strict information disclosure system. Through information disclosure, the public can quickly and accurately understand the business and financial status of the company. A well-functioning capital market with strong liquidity as well as a good information disclosure system will function well in its resource allocation role (Kyle, 1984 [25]; Merton, 1987 [26]; Tirole, 1993 [52]). In order to promote technological progress and realize scientific and technological achievements, the state can guide the allocation of financial institutions’ resources through macro-policies, increase financial support for energy-saving and low-carbon industries, reduce the threshold for the listing of technology-based enterprises, and alleviate the shortage of funding for technological progress. 

For an increasingly mature and well-functioning financial market, how to reduce the risks brought by technological development is a particularly important question. The financial system needs to provide a good channel for investors to achieve rapid liquidation and reduce the risk of innovative investment projects through the provision of financial asset portfolios. Portfolio products allow investors to increase the financing of otherwise illiquid projects with long payback periods, and so they alleviate the shortage of funding for technology-based long-term projects. Other areas where highly developed financial markets can support emissions reductions include innovative insurance products, such as insurance for developers of low-carbon projects, whereby compensation is awarded if the project fails to achieve the expected benefits, and the establishment of a risk compensation mechanism to provide credit guarantees to compensate any losses incurred by enterprises in the process of reducing emissions.

The financial development of a country or region is an important factor in foreign direct investment (FDI), and it also affects the technology spillover from FDI. When the level of financial development is high, more FDI is attracted, which is conducive to technological progress. Hermes and Lensink (2003) [53] believe that the developed financial system: has a good savings and investment conversion mechanism; can effectively supervise technological transformation and research and development projects invested in; provides financial support for domestic enterprises to train employees, which promote human capital accumulation; and attracts FDI, which brings new technology, improves employee quality, and increases corporate strength.

**Figure 1:**
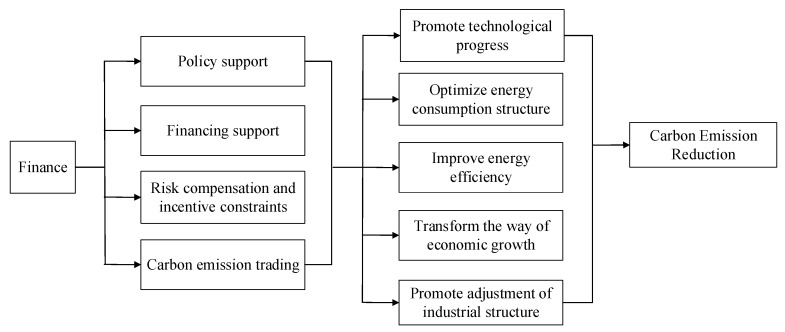
Path analysis of financial development affecting the reduction in carbon emissions.

In addition to affecting technological progress through resource allocation, risk dispersion, and impact of FDI technology spillovers, financial development also has an impact through channels that promote human capital accumulation. Human capital is one of the important factors affecting technological progress. Through the study of relevant knowledge and theoretical skills, human capital transforms knowledge and theory into scientific and technological achievements, thereby promoting technological progress. The formation of human capital depends on the continuous accumulation of knowledge. A certain amount of human capital is necessary for enterprises to promote technological improvement and achieve technological innovation. The quantity and quality of human capital are related to the education system. Generally speaking, the better a country’s or region’s education system is, the greater will be its human capital and therefore its ability to transform scientific theory into results. Therefore, its technological improvement, research and development and innovation capabilities are stronger. Technological progress will promote reductions in carbon emissions by improving energy efficiency, optimizing the structure of energy consumption, and optimizing industrial structure. Therefore, this paper further analyzes how financial development affect reduction in carbon emission in high-energy industries, as shown in Figure 1.

As can be seen from Figure 1, financial development can promote carbon emission reduction through various channels, including financial policy transmission mechanisms, financing mechanisms, risk compensation mechanisms, and carbon emission trading mechanisms. Financial policy support for low-carbon development can achieve its objectives based on policy instruments, such as credit and preferential interest rates, different credit lines, and loan interest rates, are given to enterprises according to their energy consumption and emissions, to promote enterprises to optimize energy consumption structure and improve energy efficiency. By providing financing for emission reduction projects and technologies through various channels such as credit market, stock market, and funds, the resource allocation function of financial institutions can be effectively utilized. The development of low-carbon technology has great risks. By establishing a risk compensation mechanism, providing credit guarantee for enterprises and setting up a carbon risk fund, it can transfer the risks faced by the development and production of low-carbon technology to some extent. The establishment of a carbon emission trading mechanism enables companies to independently choose to reduce emissions or purchase emission rights, and increase the power of emission reduction. Therefore, financial development can effectively promote the development and promotion of low-carbon technologies, improve energy efficiency. By providing supports, such as low-interest loans, it can effectively promote enterprises to optimize energy consumption structure, transform the way of economic growth, and adjust the industrial structure, and ultimately help to achieve carbon emission reduction.

## 4. Specification of the Theoretical Model

The traditional linear regression model only studies the stability relationship between variables, and does not consider the possible nonlinear relationship between economic variables. When there is a threshold effect, some researchers will subjectively determine one or more threshold values based on experience and descriptive analysis of the data, and then segment the sample with these threshold values, and will not do parameter estimation and significance test for the threshold values. For example, when Han et al. (2009) [54] studied the relationship between economic growth and the environment in different countries, they made an artificial determination of the income level and industrial level of the countries in the sample, and divided the total sample into four groups. However, the differentiation intervals and thresholds of this exogenous grouping method are arbitrarily chosen, rather than determined by the economic internal mechanism. Therefore, the grouping results may have strong subjectivity, and the conclusions have a strong correlation with the selection of grouping criteria. Tong (1980) [55] first proposed the threshold auto-regression model, which is widely used in economics and finance, and it can avoid the bias caused by thresholds by subjectively determined. Hansen (1999) [56] proposed a static panel threshold model, which extended the threshold analysis from time series data analysis to panel data analysis, and proposed a thresholds estimation method. In the test of whether there is a threshold effect, the distribution of test statistic is non-standard due to the existence of unknown parameters. Hansen uses the bootstrap method to calculate the progressive distribution of test statistic, which greatly compensates the shortage of the previous research.

The main idea of panel threshold model is to add the threshold value as an unknown variable to the general measurement model, and construct a piecewise function of explanatory variables. To analyze the impact of financial development on carbon emissions, the panel-threshold model introduced by Hansen (1999) [56] was used. The single-threshold model is set as follows:

(1) $$y_{it}=u_{t}+\propto Z_{it}+\beta_{1}x_{it}I\left(q_{it}\leq \gamma \right)+\beta_{2}x_{it}\left(q_{it}>\gamma \right)+\varepsilon_{it}$$

where $y_{it}$ and $x_{it}$ are explained variables and explanatory variables respectively, subscripts *i* and *t* represent “individual” variables and “time” variables respectively, individual effects are represented by $u_{t}$, and $\varepsilon_{it}$ is random interference term, where $\varepsilon_{it}$~iidN(0,σ^2^). $Z_{it}$ is the controlled variable and ∝ is the corresponding coefficient vector of each controlled variable, $\beta_{1}$ and $\beta_{2}$ are regression coefficients, $q_{it}$ is the threshold variable, and γ is the specific threshold value. I( ) is an indicative function, whose value is 0 or 1: when the corresponding conditions are established, the value is 1; when they are not established, the value is 0. When the threshold variable is in different intervals separated by specific threshold values, the degree of influence of the explanatory variable on the explained variable is different, which shows as a difference in the regression coefficient on the explanatory variable. Therefore, Equation (1) can be expressed intuitively as follows:

(2) $$y_{it}=\{\begin{array}{c} u_{t}+\propto Z_{it}+\beta_{1}x_{it}+\varepsilon_{it},q_{it}\leq \gamma \\ u_{t}+\propto Z_{it}+\beta_{2}x_{it}+\varepsilon_{it},q_{it}>\gamma \end{array}$$

### 4.1. Model Estimation

When building a threshold model for analysis, it is necessary to estimate the model. The first step is to determine the estimated value of the parameter. To eliminate the influence of individual effects, the average value of each group is subtracted from each observed value. Equation (1) can be transformed to obtain:

(3) $$y_{it}^{*}=u_{t}^{*}+\propto Z_{it}^{*}+\beta_{1}x_{it}^{*}I\left(q_{it}\leq \gamma \right)+\beta_{2}x_{it}^{*}I\left(q_{it}>\gamma \right)+\varepsilon_{it}^{*}$$

where:

(4) $$\{\begin{array}{c} y_{it}^{*}=y_{it}-\bar{y_{i}}=y_{it}-\frac{1}{T}\overset{T}{\underset{t=1}{\sum }}y_{it}\\ x_{it}^{*}\left(\gamma \right)=x_{it}\left(\gamma \right)-\bar{x_{i}}\left(\gamma \right)=x_{it}\left(\gamma \right)-\frac{1}{T}\overset{T}{\underset{t=1}{\sum }}x_{it}\left(\gamma \right)\\ e_{it}^{*}=e_{it}-\bar{e_{i}}=e_{it}-\frac{1}{T}\overset{T}{\underset{t=1}{\sum }}e_{it} \end{array}$$

Further, all individual observed values are stacked and Equation (2) is transformed into:

(5) $$Y^{*}=X^{*}\left(\gamma \right)\beta +\varepsilon^{*}$$

For a given threshold value γ, the OLS method is used for the estimations. The estimated value of β is obtained as follows:

(6) $$\beta (\gamma )={\left(X^{*}{\left(\gamma \right)}^{\prime }X^{*}\left(\gamma \right)\right)}^{-1}X^{*}{\left(\gamma \right)}^{\prime }Y^{*}$$

The corresponding residual vectors are:

(7) $${\hat{e}}^{*}\left(\gamma \right)=Y^{*}-X^{*}\left(\gamma \right)\cdot \beta^{*}\left(\gamma \right)$$

The sum of squares of residuals is:

(8) $$S_{1}\left(\gamma \right)={\hat{e}}^{*}{\left(\gamma \right)}^{\prime }{\hat{e}}^{*}\left(\gamma \right)$$

The threshold value, γ, is estimated by the least-squares method, that is, the γ value when the sum of the squares of the residuals is smallest. $\hat{\gamma }=argminS_{1}\left(\gamma \right)$. Then we can get the estimated value of β, that is, $\beta =\beta \left(\hat{\gamma }\right)$, and the estimated value of residual vector is ${\hat{e}}^{*}={\hat{e}}^{*}\left(\gamma \right)$.

### 4.2. Hypothesis Testing

#### 4.2.1. Threshold Effect Test

When setting up the model, it is assumed that there is a threshold effect (this is the hypothesis), but whether it is statistically significant needs to be tested. The null hypothesis is that there is no threshold effect:

$$H_{0}:\beta_{1}=\beta_{2}$$

The alternative hypothesis is:

$$H_{1}:\beta_{1}\neq \beta_{2}$$

The test statistic is:

(9) $$F_{1}=\frac{S_{0}-S_{1}\left(\hat{\gamma }\right)}{{\hat{\sigma }}^{2}}$$

Under the null hypothesis, there is no threshold effect, the threshold value, γ, cannot be recognized, and the distribution of the traditional F test statistic is non-standard. Hansen (1999) [56] proposed to simulate its asymptotic distribution by a “bootstrap” method to construct its *p* value. If its *p* value is less than the selected significance level, the null hypothesis is rejected and the threshold effect is demonstrated.

#### 4.2.2. Asymptotic Distribution Characteristics of Threshold Estimates

Under the premise of a significant threshold effect, the confidence interval for the threshold value is constructed by likelihood ratio statistics to test whether the threshold estimate is equal to the true value. The null hypothesis is:

$$H_{0}:\hat{r}=r_{0}$$

The likelihood ratio statistic is:

(10) $${\mathrm{LR}}_{1}\left(\gamma \right)=\frac{S_{1}\left(\gamma \right)-S_{1}\left(\hat{\gamma }\right)}{{\hat{\gamma }}^{2}}$$

The distribution of the likelihood ratio statistic is non-standard. Hansen (1999) [56] pointed out that at a given significance level, ∝, when LR_1_(γ_0_) > C(α), (where $C\left(\propto \right)=-2\ln\left(1-\sqrt{1-\propto }\right)$), the null hypothesis is rejected. 

In the model specification, it is assumed that there is only one threshold, but often there are many thresholds. The multiple-threshold test is similar to the single-threshold test. Firstly, the multiple-threshold model is estimated to determine its parameter estimates, and then the hypothesis test is conducted, including the significance test for the threshold effect and the confidence interval for the threshold parameters. 

## 5. Empirical Analysis

### 5.1. Source and Description of Data

Fossil fuels account for a large proportion of China’s energy consumption, while clean energy accounts for a small proportion. Therefore, in calculating the emissions of carbon dioxide from high-energy industries, this paper chooses nine main fossil fuels (raw coal, coke, crude oil, gasoline, kerosene, diesel, fuel oil, liquefied petroleum gas, natural gas) as the basis for consumption. Data on energy consumption come from the *China Energy Statistics Yearbooks* for the period 2005–2017. The method provided by the United Nations Intergovernmental Panel on Climate Change (IPCC) is used to estimate the carbon dioxide emissions of six high-energy industries over the period 2004–2016, namely: (1) chemical raw materials and chemicals manufacturing; (2) non-metallic mineral products; (3) ferrous metals smelting and calendaring; (4) petroleum processing; coking and nuclear fuel processing; (5) non-ferrous metals smelting and calendaring; and (6) production and supply of electricity and thermal power.

Panel data for national high-energy industries over the period 2004 to 2016 are used, and the data are selected from the annual data. The dependent variable is the logarithm of carbon dioxide emissions (lnce). Financial support is the basis for enterprises to achieve carbon emission reduction. Enterprises can obtain the funds needed for development through direct financing, or through indirect financing. In addition, theoretical analysis shows that FDI has a technology spillover effect. Foreign direct investment can not only increase financing support for industrial development but, more importantly, it can transfer technology at a lower cost and promote carbon emission reduction in high-energy-consuming industries. Therefore, for financial development, the indicators are mainly selected from three aspects: loan size (li), foreign investment (fi), and stock market value (mc). The loan size (li) is the ratio of bank loans to industrial added value in high-energy industries, data come from the China Energy Statistics Yearbooks. The foreign investment (fi) is the proportion of foreign investment in fixed asset investment in high-energy industries, data come from the China Energy Statistics Yearbooks. The stock market value (mc) is the ratio of the stock market value to the industrial added value. The stock market value data come from the “RESSET” database. One threshold variable is a technological indicator, namely R and D expenditure (rd). In addition, energy intensity (ratio of energy consumption to industrial added value), energy structure (the proportion of raw coal terminal consumption to energy terminal consumption) and economic structure (the industrial added value of each high-energy industries accounts for the proportion of GDP) are also important factors affecting carbon dioxide emissions. Therefore, energy intensity (ci), energy structure (cp), and economic structure (is) are also selected as threshold variables. Descriptive statistics of variables are shown in Table 1.

### 5.2. Empirical Results and Analysis

The panel-threshold model requires that the variables be stationary series. Therefore, before analyzing the panel-threshold effect, the stationarity test of the variables was performed. Im–Pesaran–Shin test (IPS), Augmented Dickey-Full test (ADF) and Phillips-Perron Fisher test (PP) were used. According to the results of unit root test, under the three test methods, each variable rejects the null hypothesis of unit root at the 5% significance level, which indicates a stationary series (Table 2). Therefore, a panel-threshold model can be constructed. The panel-threshold effect can be analyzed using Stata 12.0 software (StataCorp LLC, College Station, TX, USA).

#### 5.2.1. Panel-Threshold Effect of Loan Size on Carbon Emission Reduction in High-Energy Industries

(1) Threshold estimation

In setting up the model, it is assumed that there is a threshold effect, so before analysis, it is first necessary to check whether there is a threshold effect in the relationship between loan size and carbon dioxide emissions, and then to determine the number of thresholds. Based on the threshold effect test method proposed by Hansen (1999) [56], and taking loan size as the core explanatory variable, the R and D expenditure, energy intensity, energy structure and economic structure variables are used as threshold variables to estimate the model. The threshold effect test results are shown in Table 3 for the case that only one threshold exists, two thresholds exist, and three thresholds exist. 

Table 3 shows that when R and D expenditure (rd) is the threshold variable in the model constructed with loan size (li) as the core explanatory variable, under the null hypothesis that there is one threshold, the value of the F statistic is 26.438, which passes the 5% significance test. The F-value of the double-threshold model is 9.505, which rejects the null hypothesis of the two thresholds at the 5% significance level, so there is one threshold. When energy intensity (ci) is taken as threshold variable, the F-value of the single-threshold model is 17.756, which is significant at the 5% level. The F-value of the double-threshold model is 3.578, which fails to pass the 5% significance test. Therefore, there is one threshold. With energy structure (cp) as the threshold variable, the single-threshold model is significant at the 5% level, the F-value of the double-threshold model is 7.542, and the null hypothesis of two threshold values is rejected at the 5% significance level, so there is one threshold. When the economic structure (is) is taken as the threshold variable, the F-value of the double-threshold model is 12.691, which is significant at the 5% level, but the three-threshold model fails to pass the test at the 5% significance level. Therefore, the null hypothesis that there are two thresholds is accepted. 

**Figure 2:**
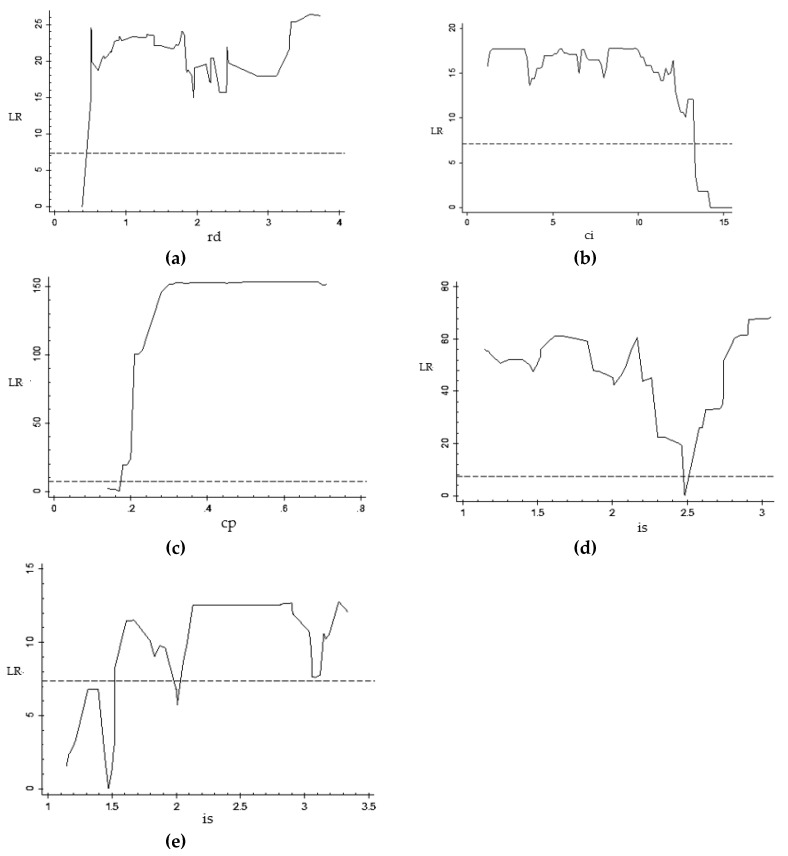
Likelihood ratio value and threshold parameter diagram of loan size and carbon emissions. (**a**) rd-single-threshold model; (**b**) ci-single-threshold model; (**c**) cp-single-threshold model; (**d**) is-single-threshold model; (**e**) is-double-threshold model.

Figure 2 shows the relationship between the likelihood ratio (LR) value and the threshold parameters. The horizontal dashed line is the critical value of the 5% significance level. The threshold parameter of the LR curve below the critical value is the confidence interval of the threshold value, and the threshold estimate is the minimum value of LR in the confidence interval.

Table 4 shows the estimates of the threshold value and the confidence intervals of each model. In the model constructed with loan size (li) as the core explanatory variable, when R and D expenditure (rd) is the threshold variable, the estimate of the threshold is 0.387, and the 95% confidence interval is [0.387, 0.387]. When energy intensity (ci) is used as the threshold variable, the threshold estimate is 14.206, and the 95% confidence interval is [13.337, 15.510]. When energy structure (cp) is the threshold variable, the threshold estimate is 0.17, and the 95% confidence interval is [0.140, 0.170]. Finally, when the economic structure (is) is taken as the threshold variable, the two threshold estimates are 2.482 and 1.472. The 95% confidence intervals are [2.482, 2.482] and [1.146, 2.008]. The two confidence intervals do not overlap, and the threshold value is significant within its confidence interval.

(2) Estimation and analysis of the threshold model

According to the test results for the threshold value, in order to measure the impact of financial development on carbon emissions in high-energy industries, a single-threshold model is constructed with loan size (li) as the core explanatory variable, and with R and D expenditure (rd), energy intensity (ci) and energy structure (cp) as the threshold variables; and a double-threshold model is constructed with economic structure (is) as the threshold variable. The estimated results are shown in Table 5. 

From the regression results of the threshold model, it can be seen that in the model constructed with R and D expenditure (rd) as the threshold variable, loan size (li) is negatively correlated with carbon dioxide emissions (lnce), indicating that loan size has a negative and indirect effect on carbon dioxide emissions, and its negative effect varies with the different intervals of R and D expenditure. Since the dependent variable is logarithmic and the independent variable is not logarithmic, according to Woodridge, its economic sense is interpreted as the percentage change in the dependent variable caused by one unit of change in the independent variable. When the R and D expenditure (rd) is less than 0.387, the loan size (li) will increase by 1% to promote the reduction of carbon dioxide emissions (lnce) by 1.1345%. And when the R and D expenditure (rd) is higher than 0.387, the emission reduction effect of loan size is strengthened. The loan size (li) increases by 1%, the carbon dioxide emissions (lnce) will be reduced by 2.5856%. These results show that the impact of loan size on carbon dioxide emissions is significantly based on the threshold effect of technological progress, and the magnitude and direction of the impact of loan size on carbon dioxide emissions depends on the level of technological progress. Despite the short sample interval in this paper, loan size has a significant emission reduction effect in the sample interval, and with the improvement of the level of technological progress and beyond a specific threshold, the emission reduction effect of loan size increases.

Technological progress is an important way to reduce emissions of carbon dioxide. The level of technological progress has been improved, low-carbon technologies have been developed, and energy utilization has been improved, thus reducing carbon dioxide emissions per unit of industrial added value. Secondly, with greater technological progress, more new products can be produced to replace energy-intensive inputs, thereby reducing carbon dioxide emissions. Wei Weixian, Yang Fang (2010) [57], and Tu Zhengge (2012) [58] show that China’s R and D expenditure has increased and technological progress has inhibitory effects on carbon dioxide emissions. At a high level of technology, higher levels of financial development are more conducive to R and D financing for enterprises, so that financial development can effectively promote reductions in carbon emissions through further technological progress. 

The empirical results also show that there is a positive V-shaped relationship between loan size (li) and carbon dioxide emissions (lnce) in the model constructed with energy intensity (ci) as the threshold variable. When the energy intensity (ci) is lower than 14.206, the relationship between loan size (li) and carbon dioxide emissions (lnce) is negative; the loan size (li) increases by 1%, the carbon dioxide emissions (lnce) are reduced by 1.3216%. When the energy intensity (ci) is higher than 14.206, the impact of loan size (li) on carbon dioxide emissions (lnce) is positive. The loan size (li) increases by 1%, the carbon dioxide emissions (lnce) increased by 12.2881%. It can be seen from the analysis that when energy intensity is high, loan size can lead to an increase in carbon dioxide emissions, but when energy intensity is below a specific threshold, loan size can promote reductions in carbon dioxide emissions. When the energy consumption per unit of industrial added value is high, the industry has low energy utilization but high emissions. The provision of financial credit promotes the development of industry and at the same time leads to an increase in carbon dioxide emissions. When energy intensity is below a specific threshold, the loan size can help to reduce carbon dioxide emissions. Mainly due to the relatively mature economic development and high level of technology, the R and D in low-carbon technology makes the energy utilization rate continuously improve. Therefore, financial development and credit support can promote reductions in carbon dioxide emissions in high-energy industries. 

In the model constructed with energy structure (cp) as threshold variable, the impact of loan size (li) on carbon dioxide emissions (lnce) is negative. When the energy structure (cp) is higher than 0.17, 1% increase in loan size (li) will reduce carbon dioxide emissions (lnce) by 1.2413%. When the energy structure (cp) is lower than 0.17, the emission reduction effect of loan size increases. The loan size (li) increases by 1%, carbon dioxide emissions (lnce) are reduced by 3.6106%. It can be seen from the analysis that when the proportion of coal consumption is low, loan size will have a significant emission reduction effect, and with a reduction in the proportion of coal consumption, the proportion of other low-carbon energy and clean energy will increase, and the emission reduction effect of loan size will increase significantly. 

In the model constructed with economic structure (is) as the threshold variable, the relationship between loan size (li) and carbon dioxide emissions (lnce) is negative. When economic structure (is) is higher than 2.482, the relationship between loan size and carbon dioxide emissions (lnce) is not significant. When the economic structure (is) is higher than 1.472 and lower than 2.482, loan size has a significant emission reduction effect. The loan size (li) increases by 1% to promote the reduction of carbon dioxide emissions (lnce) by 2.5178%. And when the economic structure is less than 1.472, the emission reduction effect of loan size increases. The loan size (li) increased by 1%, the emission reduction effect increases from 2.5178% to 5.5924%. It can be seen from the analysis that when the proportion of industrial added value in high-energy industries is high, loan size has no significant effect on emissions reductions. High-energy industries have obvious characteristics of high energy consumption, high pollution and high emissions. In the industrial production process, the demand for high-carbon energy is large. When the proportion of industrial added value is high, the energy consumption for industrial development is large, and financial development does not play a significant role in reducing emissions. With a decrease in the proportion of industrial added value in high-energy industries, the effect of loan size on emissions reductions gradually becomes significant, and the effect of emissions reductions is expanded with a decrease in the proportion of industrial added value. At this time, low-carbon technology is gradually improved, high-energy industries are gradually transformed, and an increase in loan size has a positive role in saving energy and reducing emissions. 

#### 5.2.2. Analysis of the Panel-Threshold Effect of Foreign Investment on Carbon Emission Reduction in High-Energy Industries

(1) Threshold estimation

Again, before analysis, it is first necessary to check whether there is a threshold effect in the relationship between foreign investment (fi) and carbon dioxide emissions (lnce), and to determine the number of thresholds. Taking foreign investment (fi) as the core explanatory variable, R and D expenditure (rd), energy intensity (ci) and economic structure (is) are the threshold variables to estimate the model. The threshold effect test results are shown in Table 6 for the case that only one threshold exists, two thresholds exist, and three thresholds exist. 

Table 6 shows that when R and D expenditure (rd) is the threshold variable in the model constructed with foreign investment (fi) as the core explanatory variable, under the null hypothesis that there is one threshold, the value of the F statistic is 10.947, which passes the 5% significance test. The F-value of the double-threshold model is 2.462, which rejects the null hypothesis of the two thresholds at the 5% significance level, so there is one threshold. When energy intensity (ci) is taken as the threshold variable, the F-value of the single-threshold model is 12.215, which is significant at the 5% level. The F-value of the double-threshold model is 5.084, which does not pass the 5% significance test, so there is one threshold. When economic structure (is) is taken as the threshold variable, the F-value of the double-threshold model is 4.864, which is significant at the 5% level, and the F-value of the three-threshold model is 3.409, which does not pass the test for 5% significance. Therefore, the null hypothesis that there are two thresholds is accepted. 

**Figure 3:**
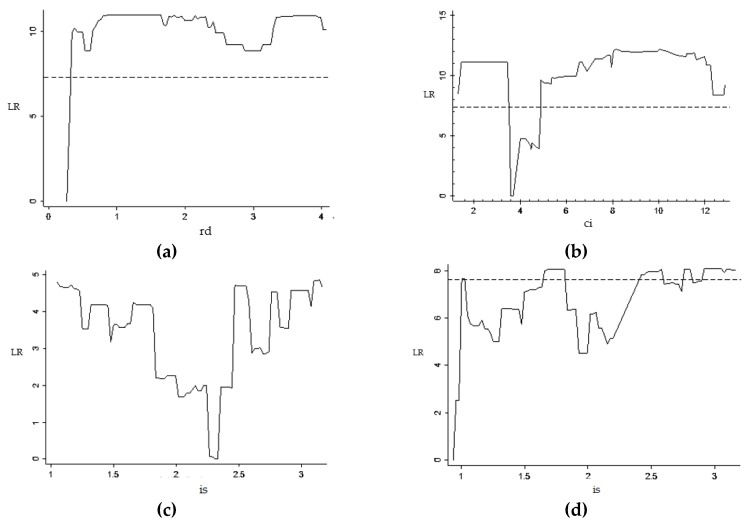
LR value and threshold parameter diagram of foreign investment and carbon emissions. (**a**) rd-single-threshold model; (**b**) ci-single-threshold model; (**c**) is-single-threshold model; (**d**) is-double-threshold model.

Table 7 shows the estimates of threshold value and the confidence intervals of each model. From Figure 3 and Table 7, it can be seen that in the model constructed with foreign investment (fi) as the core explanatory variable, when R and D expenditure (rd) is used as the threshold variable, the threshold is estimated to be 0.262. When energy intensity (ci) is used as threshold variable, the threshold estimate is 3.640. When economic structure (is) is taken as the threshold variable, the two threshold estimates are 2.301 and 0.937, and the threshold value is significant within its confidence interval.

(2) Estimation and analysis of the threshold model

According to the test results for the threshold value, in order to measure the impact of financial development on emissions reductions in high-energy industries, a single-threshold model is constructed with foreign investment (fi) as the core explanatory variable, and with R and D expenditure (rd) and energy intensity (ci) as threshold variables. Taking economic structure (is) as the threshold variable, a double-threshold model is constructed. The estimated results are shown in Table 8.

From the regression results of the threshold model, it can be seen that the relationship between foreign investment (fi) and carbon dioxide emissions (lnce) is negative in the model constructed with R and D expenditure (rd) as the threshold variable. The relationship between foreign investment and carbon dioxide emissions is affected by the level of R and D expenditure. When the R and D expenditure (rd) is less than 0.262, 1% increase in foreign investment (fi) will promote the reduction of carbon dioxide emissions (lnce) by 0.1935%. When the R and D expenditure (rd) is higher than 0.262, foreign investment (fi) increases by 1% to give a reduction in carbon dioxide emissions (lnce) of 0.0259%. The analysis shows that the impact of foreign investment on carbon dioxide emissions has a significant threshold effect based on technological progress. In an era of low R and D expenditure, the level of technological progress is relatively low. Due to its low-cost spillover effect, foreign investment can lead to the use of advanced production equipment in a relatively short time, so its role in emissions reductions is obvious. When R and D expenditure exceeds a specific threshold, the technology level at this time is relatively high. Due to the small scale of foreign investment, enterprises with foreign investment will have not introduced their advanced core technology into China, and so have little effect on emission reductions. 

The empirical results also show that the impact of foreign investment (fi) on carbon dioxide emissions (lnce) is negative in the model constructed with energy intensity (ci) as the threshold variable. When energy intensity (ci) is higher than 3.640 and foreign investment (fi) increases by 1%, carbon dioxide emissions (lnce) are reduced by 0.0499%, and when energy intensity (ci) is lower than 3.640, the effect of foreign investment (fi) on reductions in carbon dioxide emissions (lnce) increases from 0.0499% to 0.1030%. It can be seen from the analysis that foreign investment can promote the reduction of carbon dioxide emissions in high-energy industries, and this effect will increase with the reductions in energy consumption intensity. 

In the model constructed with economic structure (is) as the threshold variable, the impact of foreign investment (fi) on carbon dioxide emissions (lnce) is negative. When the economic structure (is) is higher than 2.301, carbon dioxide emissions (lnce) are reduced by 0.0467% for 1% increase in foreign investment (fi). When the economic structure (is) is higher than 0.937 and lower than 2.301, the emission reduction effect of foreign investment (fi) will be strengthened. Foreign investment (fi) increases by 1%, carbon dioxide emissions (lnce) will decrease by 0.0692%. When the economic structure is lower than 0.937, the emission reduction effect of foreign investment (fi) will be further enhanced. For 1% increase in foreign investment (fi), the carbon dioxide emissions (lnce) will be reduced by 0.1314%. According to the analysis, foreign investment has a positive role in promoting reductions in carbon emissions in high-energy industries, and this emissions reduction effect is strengthened with reductions in the proportion of industrial added value in high-energy industries.

#### 5.2.3. Analysis of the Panel-Threshold Effect of Stock Market Value on Carbon Emission Reduction in High-Energy Industries

(1) Threshold estimation

Again, it is first necessary to check whether there is a threshold effect in the relationship between the stock market value (mc) and carbon dioxide emissions (lnce), and to determine the number of thresholds. Taking stock market value (mc) as the core explanatory variable, the R and D expenditure (rd), energy intensity (ci), and energy structure (cp) are used as threshold variables to estimate the model. The results of the threshold effect test are shown in Table 9, when only one threshold exists, two thresholds exist and three thresholds exist. 

Table 9 shows that in the model with stock market value (mc) as the core explanatory variable and R and D expenditure (rd) as the threshold variable, under the null hypothesis that there is one threshold, the value of the F statistic is 21.514, which passes the 5% significance test. The F-value of the double-threshold model is 0.000, which rejects the null hypothesis of two thresholds at the 5% significance level, so there is one threshold. When energy intensity (ci) is taken as the threshold variable, the F-value of the double-threshold model is 16.297, which is significant at the 5% significance level. The F-value of the three-threshold model is 5.811, which fails to pass the 5% significance test, so there are two thresholds. With energy structure (cp) as the threshold variable, the F-value of the single-threshold model is 50.558, which is significant at the 5% level. The F-value of the double-threshold model is 1.500, which fails to pass the test at the 5% significance level. Therefore, the null hypothesis of one threshold is accepted.

**Figure 4:**
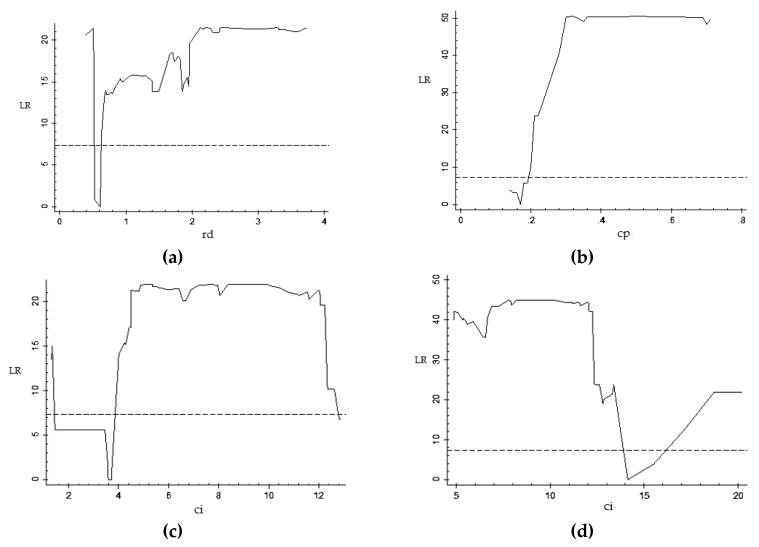
LR value and threshold parameter diagram of stock market value and carbon emissions. (**a**) rd-single-threshold model; (**b**) cp-single-threshold model; (**c**) ci-single-threshold model; (**d**) ci-double-threshold model.

Table 10 shows the estimates of the threshold value and the confidence intervals of each model. From Figure 4 and Table 9, it can be seen that in the model constructed with stock market value (mc) as the core explanatory variable, when R and D expenditure (rd) is the threshold variable, the threshold estimate is 0.610; when energy intensity (ci) is the threshold variable, the threshold estimates are 3.640 and 14.130; and when energy structure (cp) is the threshold variable, the threshold estimate is 0.170. The threshold value is significant within its confidence interval. 

(2) Estimation and analysis of the threshold model

According to the test results for the threshold value, in order to measure the impact of financial development on reduction in carbon emissions in high-energy industries, a single-threshold model is constructed with stock market value (mc) as the core explanatory variable, with R and D expenditure (rd) and energy structure (cp) as threshold variables, and a double-threshold model is constructed with energy intensity (ci) as the threshold variable. The estimated results are shown in Table 11. 

From the regression results of the threshold model, it can be seen that the relationship between stock market value (mc) and carbon dioxide emissions (lnce) is negative in the model constructed with R and D expenditure (rd) as the threshold variable, which indicates that there is a negative indirect effect of stock market value on carbon dioxide emissions. When R and D expenditure (rd) is less than 0.610 and the stock market value (mc) increases by 1%, carbon dioxide emissions (lnce) are reduced by 0.5586%. When R and D expenditure (rd) is higher than 0.610, the negative relationship between stock market value (mc) and carbon dioxide emissions (lnce) is not significant. With an increase in R and D expenditure and technological progress, stock market value does not affect emissions reductions, reflecting the imperfect development of China’s capital market. 

In the model constructed with energy intensity (ci) as the threshold variable, the relationship between stock market value (mc) and carbon dioxide emissions (lnce) is significant but V-shaped. When energy intensity (ci) is lower than 3.640, the impact of stock market value (mc) on carbon dioxide emissions (lnce) is negative, and an increase in stock market value (mc) by 1% reduces carbon dioxide emissions (lnce) by 0.5204%. When energy intensity (ci) is higher than 3.640 and lower than 14.130, the impact of stock market value (mc) on carbon dioxide emissions (lnce) is not significant. when energy intensity (ci) is higher than 14.130, the impact of stock market value (mc) on carbon dioxide emissions (lnce) becomes positive, and stock market value (mc) increases 1%, carbon dioxide emissions (lnce) are increased by 0.6662%. It can be seen from the analysis that when energy consumption intensity is high, the technology level is low, and an increase in stock market value leads to an increase in carbon dioxide emissions; in contrast, when the energy consumption intensity is low, the technology level is high, and stock market value has a positive role in reducing carbon dioxide emissions in high-energy industries. 

In the model constructed with energy structure (cp) as the threshold variable, the impact of stock market value (mc) on carbon dioxide emissions (lnce) is negative. When the energy structure (cp) is lower than 0.170, carbon dioxide emissions (lnce) decreases by 0.3807% for 1% increase in stock market value (mc); when the energy structure (cp) is higher than 0.170, the negative relationship between stock market value (mc) and carbon dioxide emission (lnce) is not significant. It can be seen from the analysis that when the proportion of coal in energy consumption is high, the carbon emission reduction effect of stock market value is not significant; however, when the proportion of coal in energy consumption is low, the stock market value can significantly promote emissions reductions in high-energy industries.

## 6. Conclusions

Since the impact of financial development on carbon emissions is bidirectional, it cannot only increase carbon dioxide emissions by promoting the expansion of the economy, but it must also have a technological and structural effect to promote reductions in carbon emissions. Therefore, this paper uses a panel-threshold model and takes technological progress, energy intensity, energy structure, and economic structure as threshold variables. Viewing financial development in terms of loan size, foreign investment, and stock market value, the paper analyzes whether there is a threshold effect of financial development on carbon emission reduction in high-energy industries, as well as the impact of financial development on reductions in carbon emissions in high-energy industries when the threshold variables are within different intervals. The research findings show that there is a threshold effect in the impact of financial development on carbon dioxide emissions. When the threshold variables are within different intervals, the impact of financial development on carbon dioxide emissions is different. From the perspective of emissions reductions, the effect of loan size is the strongest, while the effect of stock market value and foreign investment is weak. From the perspective of technological progress, the carbon emission reduction effect of loan size increases with the improvement of technology, while the carbon emission reduction effect of foreign investment decreases with the improvement of technology. Stock market value can play a significant role in reducing emissions at a low level of technological development, while at a high level of technological development its role is not significant. This reflects the fact that China’s capital market needs further improvement before it can achieve better resource allocation. 

Theoretical analysis and empirical results show that financial development can effectively promote carbon emission reduction, so it is necessary to increase financing support in the field of carbon emission reduction. Since emission reduction projects usually have large capital expenditures, long-term effects, and a lack of guarantees and mortgages, commercial banks have not maintained a positive attitude on the credit application of enterprises to apply for emission reduction projects for many years. However, as the low-carbon economy becomes a hot spot for China’s industrial sustainable development, more and more banks are beginning to provide financial support for the environmental protection field. China’s “Recommendations for the 13th five-year plan for economic and social development” proposed “effectively controlling carbon emissions in key industries such as electricity, steel, building materials, and chemicals, and promoting low-carbon development in key areas such as industry, energy, construction, and transportation.” It reflects the country attaching great importance to low carbon development, and it points out some measures to promote low-carbon development, such as “increase the promotion of low-carbon technologies and products”; “establish a green financial system, develop green credit, green bonds, and establish a green development fund”; “encourage foreign investment to invest more in advanced manufacturing, high-tech, energy-saving and environmental protection, modern service industries and the Midwest and Northeast China, and support the establishment of research and development centers”. It can be seen that the state actively guides the society to provide financial support for the low-carbon sector and promotes the development and promotion of low-carbon technologies. Therefore, it is still necessary to increase financial support for carbon emission reduction at this stage. 

Based on the perspective of technological progress, this paper analyzes the impacts of financial development on carbon emission reduction in high-energy industries. The impacts are complex and multifaceted, and further research is needed. Since financial development cannot only promote the reduction of carbon emissions through technological advancement, but also may directly promote the increase of carbon emissions through scale effects, etc., it is necessary to analyze the direct effects and scale effects of financial development on carbon emissions in high-energy industries in the future.

## Figures and Tables

**Table 1:** Descriptive statistics.

Variable	Observation	Mean	Standard Deviation	Min	Max
lnce	78	9.7459	1.3022	7.5494	11.8146
li	78	0.1186	0.1236	0.0257	0.5327
fi	78	2.4684	2.8038	0.0974	13.1898
mc	78	0.5312	0.2904	0.0932	1.5009
rd	78	1.8772	1.2562	0.2	4.3452
ci	78	7.7374	4.8825	1.0603	20.9892
cp	78	0.3275	0.2183	0.0912	0.8284
is	78	2.0980	0.7720	0.673	3.651

**Table 2:** Unit root test results.

Test Methods	lnce	li	fi	mc	rd	ci	cp	is
Im–Pesaran–Shin	−9.6188 ***	−2.9946 ***	−2.4410 ***	−4.2606 ***	−32.9293 ***	−30.8276 ***	−5.0938 ***	−6.1442 ***
	(0.0000)	(0.0014)	(0.0073)	(0.0000)	(0.0000)	(0.0000)	(0.0000)	(0.0000)
Augmented Dickey-Ful	62.5004 ***	31.3095 ***	25.3356 **	38.4402 ***	36.0072 ***	48.7172 ***	44.3255 ***	52.6615 ***
	(0.0000)	(0.0018)	(0.0133)	(0.0001)	(0.0003)	(0.0000)	(0.0000)	(0.0000)
Phillips-Perron Fisher	66.2498 ***	29.9475 ***	21.4477 **	43.4099 ***	52.1090 ***	45.2106 ***	57.3793 ***	52.0816 ***
	(0.0000)	(0.0028)	(0.0442)	(0.0000)	(0.0000)	(0.0000)	(0.0000)	(0.0000)

Note: () is t-test statistic; *** and ** are significant at 1% and 5% significance levels, respectively.

**Table 3:** Threshold effect test results of loan size and carbon emissions.

Threshold Variable	Model	F-Value	*p*-Value	Bootstrap Times	Critical Value		
					1%	5%	10%
rd	Single-Threshold Model	26.438 ***	0.007	300	24.607	14.333	8.172
	Double-Threshold Model	9.505	0.827	300	36.923	29.434	24.820
	Three-Threshold Model	7.053	0.155	200	13.702	10.252	8.344
ci	Single-Threshold Model	17.756 ***	0.000	300	7.727	4.489	3.112
	Double-Threshold Model	3.578 *	0.0620	300	7.241	3.832	2.742
	Three-Threshold Model	3.243 *	0.057	200	7.911	3.513	1.820
cp	Single-Threshold Model	152.977 ***	0.000	300	101.800	62.894	30.346
	Double-Threshold Model	7.542	0.177	300	21.450	13.981	9.848
	Three-Threshold Model	2.679	0.135	200	7.418	4.123	3.334
is	Single-Threshold Model	39.214 ***	0.010	300	37.249	23.083	16.860
	Double-Threshold Model	12.691 **	0.043	300	17.744	12.564	9.653
	Three-Threshold Model	5.932	0.110	200	12.949	7.819	6.451

Note: ***, **, * are significant at 1%, 5%, and 10% significance levels, respectively.

**Table 4:** Threshold estimation results and confidence intervals of loan size and carbon emissions.

Threshold Variable	Threshold Value	Estimates	95% Confidence Interval
rd	γ	0.387	[0.387, 0.387]
ci	γ	14.206	[13.337, 15.510]
cp	γ	0.170	[0.140, 0.170]
is	γ1	2.482	[2.482, 2.482]
	γ2	1.472	[1.146, 2.008]

**Table 5:** Threshold model regression results of loan size and carbon emissions.

Test Variable	Threshold Variable			
	rd	ci	cp	is
rd		0.1797 ***	−0.1645	0.0676
		(4.9906)	(−0.77)	(1.65)
ci	0.0788 ***		0.0731 ***	0.0511 ***
	(10.35)		(9.35)	(4.38)
cp	0.8855 ***	1.9272 ***		1.2418 ***
	(2.93)	(4.5368)		(3.02)
is	0.3163 ***	0.4262 ***	0.2491 ***	
	(7.16)	(7.7319)	(7.40)	
li_1	−1.1345 ***	−1.3216 ***	−3.6106 ***	−5.5924 ***
	(−3.21)	(−2.6830)	(−8.62)	(−5.18)
li_2	−2.5856 ***	12.2881 ***	−1.2413 ***	−2.5178 ***
	(−5.35)	(3.6818)	(−4.29)	(−4.29)
li_3				−0.6199
				(−1.33)
cons	8.4149 ***	7.9468 ***	8.8854 ***	9.0330 ***
	(39.65)	(37.84)	(88.74)	(53.77)
R^2^	0.9288	0.7518	0.9239	0.8022

Note: () is T-test statistic; *** and ** are significant at 1% and 5% significance levels, respectively.

**Table 6:** Threshold effect test results foreign investment and carbon emissions.

Threshold Variable	Model	F-Value	*p*-Value	BS Times	Critical Value		
					1%	5%	10%
rd	Single-Threshold Model	10.947 ***	0.005	300	8.575	4.575	2.785
	Double-Threshold Model	2.462	0.131	300	8.789	4.458	2.917
	Three-Threshold Model	0.822	0.345	200	7.141	3.687	2.613
ci	Single-Threshold Model	12.215 **	0.050	300	19.175	12.526	8.995
	Double-Threshold Model	5.084	0.213	300	23.800	16.708	11.404
	Three-Threshold Model	3.188	0.160	200	6.858	5.479	4.224
is	Single-Threshold Model	8.921 **	0.016	300	9.745	4.366	2.611
	Double-Threshold Model	4.864 **	0.026	300	7.308	3.764	2.866
	Three-Threshold Model	3.409 *	0.0620	200	7.853	3.869	2.745

Note: ***, **, * are significant at 1%, 5%, and 10% significance levels, respectively.

**Table 7:** Threshold estimation results and confidence intervals of foreign investment and carbon emissions.

Threshold Variable	Threshold Value	Estimates	95% Confidence Interval
rd	γ	0.262	[0.262, 0.300]
ci	γ	3.640	[3.560, 4.880]
is	γ1	2.301	[1.050, 3.165]
	γ2	0.937	[0.937, 0.982]

**Table 8:** Threshold model regression results for foreign investment and carbon emissions.

Test Variable	Threshold Variable		
	rd	ci	is
rd		0.1508 ***	0.0421
		(4.2)	(1.0580)
ci	0.0828 ***		0.0425 ***
	(9.0002)		(3.7754)
cp	2.2352 ***	3.1529 ***	2.8878 ***
	(7.1197)	(7.71)	(7.9001)
is	0.3458 ***	0.1146 *	
	(6.5569)	(1.94)	
fi_1	−0.1935 ***	−0.1030 ***	−0.1314 ***
	(−3.7922)	(−5.02)	(−5.6903)
fi_2	−0.0259 **	−0.0499 ***	−0.0692 ***
	(−2.6259)	(−4.1)	(−6.5933)
fi_3			−0.0467 ***
			(−4.1177)
cons	7.8670 ***	8.3324 ***	8.580 ***
	(43.16)	(40.51)	(55.65)
R^2^	0.7808	0.6406	0.751

Note: () is T-test statistic; ***, **, * are significant at 1%, 5% and 10% significance levels, respectively.

**Table 9:** Threshold effect test results for stock market value and carbon emissions.

Threshold Variable	Model	F-Value	*p*-Value	BS Times	Critical Value		
					1%	5%	10%
rd	Single-Threshold Model	21.514 **	0.017	300	22.067	18.211	10.747
	Double-Threshold Model	0.000	0.990	300	3.584	2.021	1.523
	Three-Threshold Model	1.918	0.375	200	16.242	7.682	5.521
ci	Single-Threshold Model	21.892 ***	0.010	300	21.601	15.540	9.215
	Double-Threshold Model	16.297 **	0.047	300	20.316	15.421	10.384
	Three-Threshold Model	5.811	0.135	200	13.692	9.761	7.404
cp	Single-Threshold Model	50.558 ***	0.000	300	24.006	15.709	12.209
	Double-Threshold Model	1.500	0.283	300	11.755	7.996	4.746
	Three-Threshold Model	1.403	0.405	200	10.029	8.133	6.219

Note: *** and ** are significant at 1%, and 5% significance levels, respectively.

**Table 10:** Threshold estimation results and confidence intervals for stock market value and carbon emissions.

Threshold Variable	Threshold Value	Estimates	95% Confidence Interval
rd	γ1	0.610	[0.525, 0.610]
ci	γ1	3.640	[1.450, 3.700]
	γ2	14.130	[14.130, 15.510]
cp	γ1	0.170	[0.140, 0.190]

**Table 11:** Threshold model regression results for stock market value and carbon emissions.

	Threshold Variable		
	rd	ci	cp
rd		0.1910 ***	−0.0413
		(6.28)	(−1.55)
ci	0.0899 ***		0.0855 ***
	(12.99)		(8.83)
cp	1.1672 ***	1.7976 ***	
	(5.09)	(5.05)	
is	0.3797 ***	0.2901 ***	0.3216 ***
	(9.87)	(5.76)	(7.93)
mc_1	−0.5586 ***	−0.5204 ***	−0.3807 ***
	(−5.10)	(−4.18)	(−7.06)
mc_2	−0.0535	0.0313	−0.0172
	(−1.02)	(0.635)	(−0.30)
mc_3		0.6662 ***	
		(3.72)	
cons	7.9595 ***	8.2252 ***	8.5501 ***
	(51.57)	(40.53)	(78.89)
R^2^	0.9105	0.7682	0.8657

Note: () is T-test statistic; *** is significant at 1% significance levels, respectively.
